# Association of the Nicotinic Receptor α7 Subunit Gene (CHRNA7) with Schizophrenia and Visual Backward Masking

**DOI:** 10.3389/fpsyt.2013.00133

**Published:** 2013-10-22

**Authors:** George Bakanidze, Maya Roinishvili, Eka Chkonia, Werner Kitzrow, Sarina Richter, Konrad Neumann, Michael H. Herzog, Andreas Brand, Imke Puls

**Affiliations:** ^1^Genetic Section, Department of Psychiatry and Psychotherapy, CCM, Charité University Medicine, Berlin, Germany; ^2^Department of Behaviour and Cognitive Functions, I. Beritashvili Institute of Physiology, Tbilisi, Georgia; ^3^Institute of Cognitive Neurosciences, Agricultural University of Georgia, Tbilisi, Georgia; ^4^Department of Psychiatry, Tbilisi State Medical University, Tbilisi, Georgia; ^5^Institute of Biometry and Clinical Epidemiology, CCM, Charite University, Berlin, Germany; ^6^Laboratory of Psychophysics, Brain Mind Institute, Ecole Polytechnique Fédérale de Lausanne (EPFL), Lausanne, Switzerland; ^7^Center for Psychiatry and Psychotherapy, Klinikum Bremen-Ost, Bremen, Germany

**Keywords:** endophenotypes, verniers, visual masking, genetics of schizophrenia, CHRNA7, nicotinic receptor

## Abstract

The nicotinic system is involved in the pathophysiology of schizophrenia. However, very little is known about its genetic basis and how it relates to clinical symptoms and potentially pharmacological intervention. Here, we investigated five single nucleotide polymorphisms (SNPs) [rs3826029] [rs2337506] [rs982574] [rs904952] [rs2337980] of the cholinergic nicotinic receptor gene, alpha 7 subunit (CHRNA7) and their association to schizophrenia. We found an association with rs904952 (*p* = 0.009) in a German sample of 224 schizophrenic patients and 224 healthy control subjects. The same trend was shown in an independent Georgian sample of 50 schizophrenic patients, 57 first order unaffected relatives, and 51 healthy controls. In addition, visual backward masking (VBM), a sensitive test for early visual information processing, was assessed in the Georgian sample. In line with prior studies, VBM performance deficits were much more pronounced in schizophrenic patients and their unaffected relatives compared to healthy controls (schizophrenic patients: 156 ms; unaffected relatives: 60 ms; healthy controls: 33 ms). VBM was strongly correlated with SNP rs904952 (H[2] = 7.3, *p* = 0.026). Our results further support the notion that changes in the nicotinic system are involved in schizophrenia and open the avenue for pharmacological intervention.

## Introduction

Schizophrenia is a severe disease affecting patients’ professional and social life. Schizophrenia is strongly influenced by genetic factors. A large variety of candidate genes have been identified in the last years with controversial results (1–4). A major reason for the contradicting findings is the large number of genetic and environmental factors contributing to the pathophysiology of schizophrenia (5).

The nicotinic system has been found to play an important role in the pathogenesis of schizophrenia (6–8). Evidence for a relationship between the cholinergic nicotinic receptor alpha 7 subunit (CHRNA7) and schizophrenia was shown by both association (9–11) and linkage studies (12–14), although with mixed results (5, 15). A deletion of 15q13.3, the chromosomal location of CHRNA7, was identified in schizophrenic patients (16–18) together with deficiencies in cholinergic transcription in the corpus callosum (7) and reductions of the receptor protein in the hippocampus, cortex, and thalamus (6, 19, 20). Schizophrenic patients are often heavy smokers, which may be a way of self medication (21–24). Although several lines of evidence point to the nicotinic system, current drugs applied in schizophrenic patients largely neglected this system.

Visual information processing is disturbed in schizophrenic patients. For example, sensorimotor gating has been found to be impaired in schizophrenic (25) and bipolar patients with psychotic mania (26). Visual backward masking (VBM) and particularly the shine-through masking paradigm are reliable methods of measuring deficits of visual processing (27–31). The shine-through paradigm showed impaired masking in schizophrenic patients and their first-degree relatives (27) compared to healthy controls. Because of this and other findings, the shine-through masking paradigm is a potential endophenotype of schizophrenia (27). We have recently argued that these deficits are not of primary visual nature but are related to deficient mechanisms of target enhancement (32). Neural activity of low contrast or briefly presented elements needs to be enhanced to counteract overwriting of subsequently presented stimuli. We postulated that the cholinergic system is one candidate of target enhancement which may be deficient in schizophrenic patients (32). These observations are in line with other studies which showed that the nicotinic system is involved in the regulation of visual information processing (33–36) including sensory gating and P50 suppression (9, 37). Interestingly, an alpha 7 receptor agonist improved both p50 gating function and cognition in schizophrenic patients (38). The cholinergic nicotinic receptor alpha 4 subunit gene (CHRNA4) has been found to modulate both auditory and visual information processing (39). For this reason, we targeted the cholinergic system and determined the influence of five single nucleotide polymorphisms (SNPs) on the diagnosis of schizophrenia in a German sample of 224 schizophrenic patients and 224 healthy controls. We further investigated these effects in a smaller Georgian sample with 50 schizophrenic patients, 57 first-degree unaffected relatives, and 51 healthy controls and determined VBM in addition. The SNPs where selected according to ABI SNP browser selection^1^.

## Methods

### Participants

Data were collected from a German sample of 224 schizophrenic patients and 224 healthy controls. All subjects were of Central European descent. Group characteristics are depicted in Table 1. The diagnosis of schizophrenia was made according to DSM-IV criteria. Severity of psychopathology was assessed by an experienced clinician (I. Puls) using the Positive and Negative Symptom Scale (PANSS), the Scale for the Assessment of Positive Symptoms (SAPS), and the Scale for the Assessment of Negative Symptoms (SANS) (40, 41). Patients were on various neuroleptic agents, including haloperidol, olanzapine, risperidone, amisulpride, quetiapine, aripiprazole, ziprasidone. Individuals with present or life time alcohol and/or drug dependency as well as other psychiatric and neurological diseases were excluded from the study. All patients were recruited at the Department of Psychiatry and Psychotherapy, Charité, in Berlin, Germany. For comparison, 224 healthy controls were recruited from the general population. All controls were free of any axis 1 and 2 or major somatic disorders.

**Table 1:** **Demographics**.

Variable	German sample			Georgian sample			
	Patients	Controls	Statistics^a^	Patients	Relatives	Controls	Statistics^a^
Participants (#)	224	224	NA	50	57	51	NA
Male (%)	50.7	58.0	2.3 (0.13)	76	45.6	60.8	10.3 (0.006)
Age (mean)	37.0	40.8	3.33 (0.07)	34	35	34	0.16 (0.8)
Education in years (mean)	–	–	–	13	14	16	7.5 (0.001)
Visual acuity (mean VA by FrACT)	–	–	–	1.5	1.7	1.7	2.5 (0.08)

*^a^ For categorical data (gender), Chi-square statistics are reported. For visual acuity, age, and education *F* statistic is shown. In study participants from Germany, only basic clinical and psychiatric information was assessed, whereas the Georgian sample was characterized in more detail*.

For evaluation of VBM, we repeated the experiment in a Georgian sample including 50 schizophrenic patients, 57 first order relatives, and 51 healthy control subjects. All subjects were of Central European descent recruited at the Asatiani Psychiatric Institute in Tbilisi, Georgia. The diagnosis of schizophrenia was made according to DSM-IV criteria. Psychopathology was assessed using SANS and SAPS by an experienced clinician (27). Individuals with alcohol and/or drug dependency, as well as other diseases that could affect the mental state of the subject were excluded from the study. All relatives and controls were free of any axis 1 and 2 disorder, which was confirmed by the Schizotypal Personality Questionnaire (SPQ).

### Procedure

The study was approved by the local ethic committees in Berlin and Tbilisi and was performed in accordance with the Declaration of Helsinki (1964). Approvals were obtained from the Georgian National Council on Bioethics (Meeting Statement of Ethical Committee of Institute of Postgraduate Medical Education and Continuous professional Development N 9/07) and the ethics committee of the University Hospital Charite, Berlin (Ethikkommission, Ethikausschuss 1 Campus Charite – Mitte, application number: EA1/115/10). All participants gave informed written consent prior to their participation. All participants received a clinical interview, evaluating the present medical state, and prior diseases. In schizophrenic patients, the degree of psychopathology was assessed. In all study participants, 30 ml of whole blood was taken for genetic analysis.

In the Georgian sample, visual acuity was determined by the Freiburg Visual Acuity Test (FrACT) for each eye separately. A value of at least 0.8 for at least one eye was required. Afterward, the shine-through masking paradigm was performed.

### The shine-through backward masking paradigm

The shine-through backward masking paradigm was performed in the Georgian sample only. Subjects observed the stimuli from a distance of 3.5 m in a room illuminated dimly by a background light (around 0.5 lx). A pixel of the screen (19′′) comprised about 18′′ (arc sec) at this distance. Stimuli were white on a black background. Stimulus luminance was 100 cd/m^2^. Refresh rate of the screen was 100 Hz. Conditions were tested in blocks of 80 trials.

#### Vernier

A vertical vernier is composed of two vertical bars which are slightly offset either to the left or right. The length of a bar was 10′ (arc min). The bars were separated by a small gap of 1′. Thus, altogether a vernier was about 21′ long. In each trial, the vernier offset direction was chosen randomly. In a binary task, observers were asked to indicate this offset direction (left/right). Errors were indicated by an auditory signal.

#### Vernier duration

**Figure 1:**
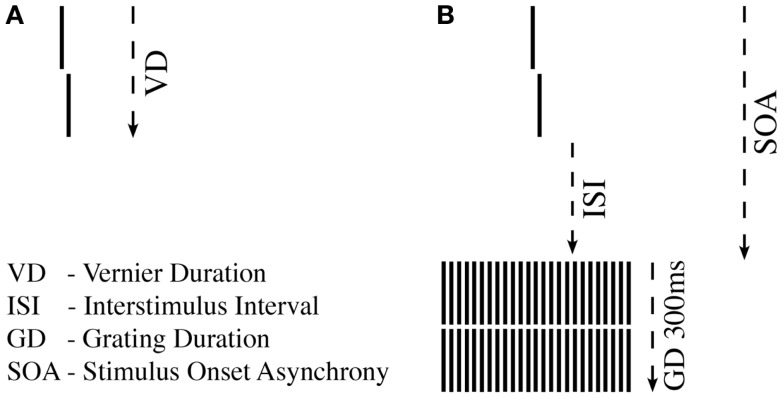
**Shine-through paradigm**. **(A)** A vernier comprised two vertical bars which were slightly offset to the left (not shown) or right (shown here). Observers indicated this offset direction. For each observer, we determined the individual vernier duration (VD) for which 75% correct responses were reached. **(B)** In the next step, this individual vernier duration was used for each observer. The vernier offset was fixed at 71′′ (arc sec). This vernier was followed by a blank screen (ISI) and a grating comprised of 25 aligned verniers, i.e., verniers without offset. SOA = VD + ISI.

First, we tested unmasked verniers, i.e., without a grating. We aimed to find the shortest VD, for which observers could perform vernier offset discrimination reliably (43) (Figure 1A). For a given VD, we determined adaptively (44) whether offset discrimination was below 40′′ (arc sec). We started with VDs of 150 ms and reduced durations blockwise until offset discrimination was above 40′′. Observers with VDs longer than 100 ms were excluded at this stage to ensure that all observers were rather “good” performers (sensitivity should not be boosted by bad “performers”).

#### Masking

In the masking experiment, we presented the vernier with the individual duration for each observer. Vernier offset size was set to 71′′. First, an inter-stimulus interval (ISI) followed the vernier, and then, a masking grating (Figure 1B). The grating comprised 25 aligned verniers, i.e., without offset, of the same length and width as the target vernier. The horizontal distance between grating elements was about 3.33′. The vernier and the central element of the grating appeared always in the middle of the screen. The grating lasted for 300 ms. We adaptively assessed the target-mask stimulus onset asynchrony (SOA) to yield a performance level of 75% correct responses for the vernier (SOA = VD + ISI). In this part of the experiment, we varied the ISI, not the vernier offset size. The adaptive strategy started with an SOA of 200 ms that was either decreased or increased to find the individual threshold. A value of 450 ms was recorded if observers were unable to reach 75% correct responses for an SOA of 400 ms (45). We did not measure accuracy for different SOAs as it was done in most other masking studies in schizophrenia research, but we determined performance in one block.

### Genetic analysis

For genetic analysis, approximately 30 ml EDTA blood samples were collected from each participant in both study cohorts. Genetic analysis for both chorts was performed in the Genetic Section, Department of Psychiatry and Psychotherapy, CCM, Charité University Medicine, Berlin. From the blood samples, DNA was extracted using standard procedures. Five SNPs of the cholinergic nicotine receptor gene, alpha 7 subunit (CHRNA7) were genotyped in the German and Georgian samples (for SNP details see Table 2). All SNPs have tagging function selected in consideration of both genetic and physical coverage that was based on Applied Biosystems SNPbrowser™ Software v3.5. Information about physical location and heterozygosity index of SNPs was derived from the University of California Santa Cruz (UCSC) Human Genome Browser^2^ and the National Center of Biotechnology Information (NCBI) SNP database^3^. Primers were designed for amplification of relevant DNA regions by polymerase chain reaction (PCR). PCR products were cut by allele specific restriction enzymes and visualized after gel electrophoresis (for PCR details see Table 3). Quality control was performed by in-gel standards to confirm expected fragments sizes and by random duplication of control samples for each plate. Primer information and specific assay conditions are available on request. Interpretation of gel electrophoresis was done by two independent investigators.

**Table 2:** **Single nucleotide polymorphisms genotyped within the CHRNA7 gene**.

CHRNA7 SNPs	Position	MAF	Alleles	Location	Reference
rs3826029	3112042	0.409	A/G	5′near gene	Iwata et al. (42)
rs2337506	3136624	0.455	G/A	Intron 2	Iwata et al. (42)
rs982574	3164580	0.215	G/C	Intron 2	Iwata et al. (42)
rs904952	3209302	0.382	C/T	Intron 4	Iwata et al. (42)
rs2337980	3234753	0.416	T/C	Intron 4	Iwata et al. (42)

*The characteristics of SNPs are derived from the NCBI database. Contig position indicates the exact position of each SNP at contig NT_010194.16/17. MAF (minor allele frequency) is derived from 1000 Genome phase 1 genotype data from 1094 worldwide individuals, released in the May 2011 dataset. The minor allele is depicted in the first position. Location indicates the SNP position within the CHRNA7 gene*.

**Table 3:** **Amplification and restriction details of SNPs studied**.

CHRNA7 SNPs	Genotype	Forward primer	Reverse primer	Product size	Restriction products	Enzyme	*t*°	GFR in%	
								DE	GEO
rs3826029	A/G	gtgaatccagttcagctgtc	gtgactgagcttgactgtac	243	243, 137, 106	*Afl*II	56	0.4	0
rs2337506	G/A	gccttggagtcacagctc	gcaatttcctactcctcgtc	255	255, 170, 85	*Bss*SI	56	0.4	0
rs982574	G/C	cataacttagaatactcaacaag	gttgccaccacatctaccttc	164	164, 86, 78	*Mnl*I	56	0.9	0.6
rs904952	C/T	caaattggttaatttctgttcctAg	ccatggaaaacaggatgagtg	134	134, 109, 25	*Alu*I	56	0	0
rs2337980	T/C	ctgtcctccggtatctgtg	cagtcacttctgtgtctaag	271	(193, 78), 156, 37	*Bsr*I	56	0.2	0.6

*Forward and reverse primers, as well as product sizes, restriction products, and restriction enzymes are shown in the table. In a forward primer of rs904952 C has been mutated into A, it is shown as captial letter. *Bsr*I was a double cutter, therefore additional restriction product emerged (shown in brackets). *t*°, annealing temperature; GFR, genotyping fail rate; DE, German sample; GEO, Georgian sample*.

### Statistical analysis

**Figure 2:**
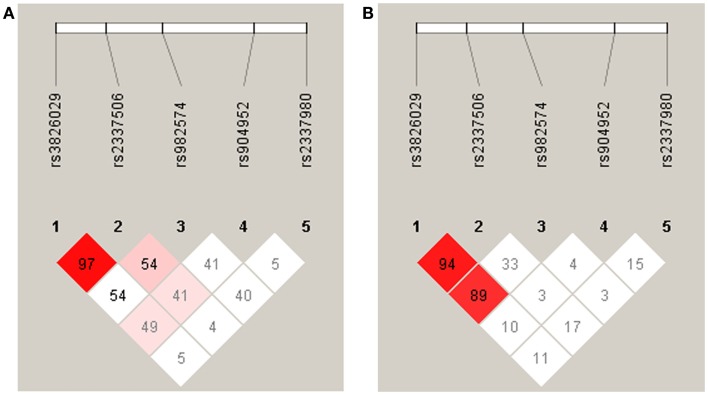
**Linkage disequilibrium analysis in German (A) and Georgian (B) samples**. The strength of LD is determined as Lewontin’s D′. In the German sample the SNPs rs3826029 and rs2337506 showed high linkage with D′ of 0.97. In the Georgian sample, rs3826029 and rs2337506 with D′0.94 and rs3826029 and rs982574 with D′ of 0.89 were in high linkage disequilibrium.

For statistical analysis, SPSS v. 16.0 was used. We measured the association of genotype and diagnosis on both samples. In the Georgian sample, we additionally measured the SOA differences between the groups and the differences of SOA’s between the carriers of different genotypes. To test for a relationship between categorical variables, such as allele variants (three categories) and study groups (three categories: patients, relatives, and controls), a crosstabulation analysis using Chi-square statistics was applied. For the analysis of categorical data such as gender, the Chi-square test was used. For quantitative, normally distributed data, *F* statistics was used. For quantitative, non-normally distributed data [like shine-through data (SOA)] Kruskal–Wallis and Mann–Whitney tests were used. The power analysis was conducted using software G*Power version 3.1.3 (46). We expected an effect size of approximately 0.3 (Cramer’s V). A sample size of *n* = 448 is sufficiently large to detect an effect size of 0.3 (Cramer’s V) with a power larger than 80% (1-beta = 0.858) assuming alpha = 0.01. Hardy–Weinberg equilibrium (HWE) and linkage disequilibrium (LD) were calculated with Haploview version 4.2 (47, 48). Details on HWE, as well as allele and genotype frequencies for each sample are given in Tables 4, 5, and 6. Details on LD are depicted in Figures 2A,B. Tagging SNPs were defined by Applied Biosystems SNPbrowser™ Software v3.5.

**Table 4:** **Hardy–Weinberg equilibrium in German and Georgian samples**.

SNP	Genotype	HWE *p*-value	
		German	Georgian
rs3826029	A/G	0.578	0.377
rs2337506	G/A	0.899	0.946
rs982574	G/C	0.146	1.0
rs904952	C/T	0.005	0.632
rs2337980	T/C	0.566	0.513

**Table 5:** **Genotype frequencies in German and Georgian samples**.

	rs3826029			rs2337506			rs982574			rs904952			rs2337980		
	A/A	A/G	G/G	G/G	G/A	A/A	G/G	G/C	C/C	C/C	C/T	T/T	T/T	T/C	C/C
**GEORGIAN**															
Whole	0.089	0.361	0.551	0.449	0.437	0.114	0.025	0.266	0.703	0.127	0.494	0.380	0.367	0.500	0.127
Patients	0.040	0.360	0.600	0.480	0.460	0.060	0.060	0.240	0.700	0.060	0.560	0.380	0.480	0.400	0.120
Relatives	0.070	0.421	0.509	0.456	0.439	0.105	0.018	0.246	0.719	0.088	0.491	0.421	0.351	0.474	0.175
Controls	0.157	0.294	0.549	0.412	0.412	0.176	0	0.314	0.686	0.235	0.431	0.333	0.275	0.627	0.078
**GERMAN**															
Whole	0.025	0.295	0.676	0.563	0.375	0.058	0.040	0.210	0.777	0.201	0.567	0.232	0.225	0.513	0.259
Patients	0.022	0.317	0.661	0.558	0.393	0.049	0.040	0.246	0.741	0.152	0.638	0.210	0.210	0.554	0.237
Controls	0.027	0.272	0.692	0.567	0.357	0.067	0.040	0.174	0.813	0.250	0.496	0.254	0.241	0.473	0.281

*Genotype frequencies for each SNP in both samples and in each group of these sample*.

**Table 6:** **Association of CHRNA7 with diagnosis in German and Georgian samples**.

	Minor/major	Georgian sample			χ^2^	*p*-Value	German sample		χ^2^	*p*-Value
		MAF					MAF			
		Patients	Relatives	Controls			Patients	Controls	
rs3826029	A/G	0.220	0.281	0.304	5.84	0.212	0.181	0.164	1	0.606
rs2337506	G/A	0.290	0.325	0.382	3.47	0.482	0.246	0.248	1	0.606
rs982574	G/C	0.180	0.143	0.157	4.46	0.347	0.128	0.092	3.46	0.177
rs904952	C/T	0.340	0.333	0.451	8.85	0.072	0.471	0.495	6.73	0.009
rs2337980	T/C	0.320	0.412	0.400	7.74	0.101	0.487	0.477	2.75	0.252

*Association of CHRNA7 polymorphisms with the diagnosis in German and Georgian samples. Showing the genotype with minor allele in the first position, MAF, minor allele frequencies; χ^2^, Chi-square value; *p*, significance*.

## Results

### Demographics

In the German sample, age (*p* = 0.069) and gender (*p* = 0.129) did not differ significantly between controls and patients. In the Georgian sample, groups also did not differ significantly for age (*F*[2,155] = 0.168; *p* = 0.8). But gender [χ^2^(2) = 10.3; *p* = 0.006] and education (*F*[2,155] = 7.5; *p* = 0.001) were significantly different in the Georgian sample, with fewer females in the patient group and patients having poorer education compared to controls (Table 1). However the difference between males and females on VBM performance did not differ significantly (*U* = 208.5, *p* = 0.66).

### CHRNA7 and the diagnosis of schizophrenia

**Figure 3:**
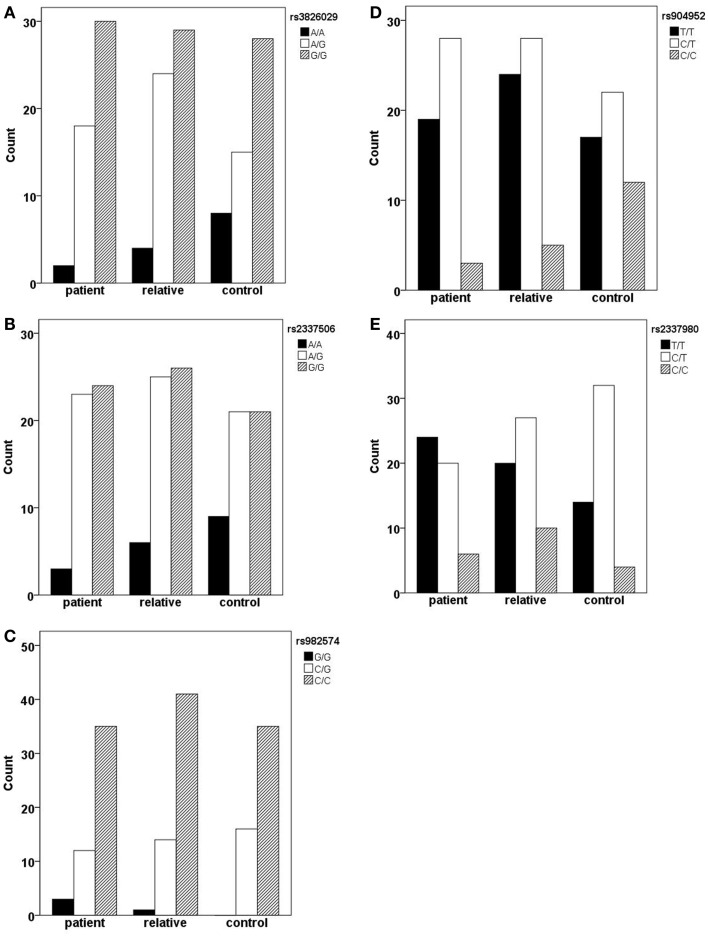
**Allelic variants of the five SNPs (rs3826029 (A), rs904952 (B), rs2337506 (C), rs2337980 (D), rs982574 (E)) of patients, first-order relatives, and healthy controls**. In all of the five SNPs, for at least one allelic variant the count for relatives is in between patients and controls. However, the differences between groups are not statistically significant.

In the German sample, we found an association of CHRNA7 with the diagnosis of schizophrenia. In SNP rs904952, the T allele was more frequently expressed in schizophrenic patients compared to controls [χ^2^(1) = 6.37; *p* = 0.009] (Table 6). The *p*-value remained significant after Bonferroni correction for five SNPs (0.05/5 = 0.01). In the Georgian sample, a relation between genetic variants in the CHRNA7 gene and the diagnosis of schizophrenia was found. In all of the five SNPs, including rs904952 and rs2337980 located in intron 4 of the CHRNA7 gene, the distribution of at least one allelic variant showed a pattern were relatives lie in between of patients and controls. The differences between the groups, however, did not reach significance likely because of the small sample size (Table 6; Figures 3A–E).

### Stimulus onset asynchrony

**Figure 4:**
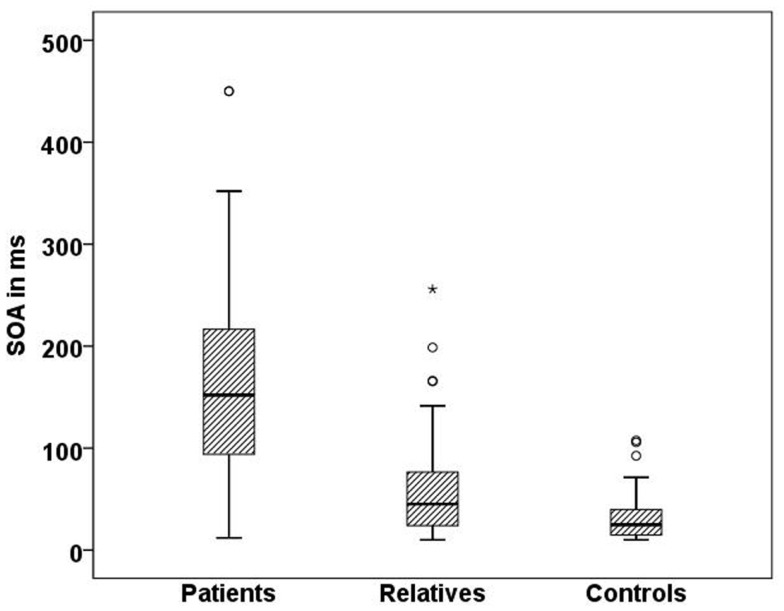
**Mean SOA in milliseconds for each Georgian group**. Schizophrenic patients (*n* = 50) perform worse than healthy relatives (*n* = 57) and controls (*n* = 51), SD = 81.7,*p* < 0.001.

In line with previous studies (9, 27), we found significant differences in visual masking between patients, their first order relatives, and controls in the Georgian sample with the longest SOA for schizophrenic patients (156 ms), followed by the unaffected relatives (60 ms), and controls (33 ms) (H[2] = 68.3, *p* < 0.0001; Figure 4). Within the patient group, there was no significant difference between subjects taking the anticholinergic agent trihexyphenidyl and those who did not. Effects of other neuroleptic drugs have not been evaluated because of small sizes.

### CHRNA7 and SOA

**Figure 5:**
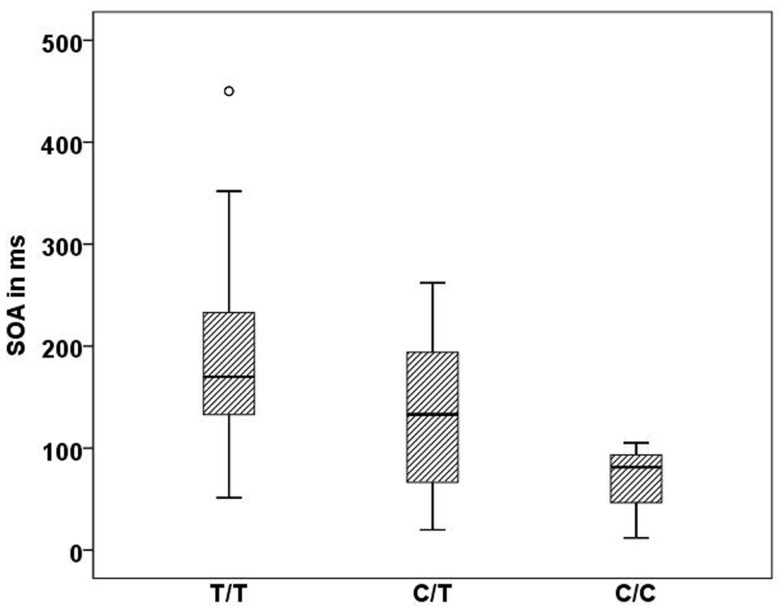
**Stimulus onset asynchrony’s of the different genotype carriers of SNP rs904952 in the Georgian patient group**. SD = 95.1,*p* = 0.026.

In the Georgian sample, we found an association of the CHRNA7 gene with SOA level, with again the T allele of rs904952 as the risk variant. In the patient group, a significant effect of rs904952 on the SOA was found (H[2] = 7.3, *p* = 0.026; Figure 5). Patients with the T/T genotype performed significantly worse than carriers of C/T and C/C. We used the Mann–Whitney test to calculate the difference between the VBM performance of T/T and C/C carriers. The difference was statistically significant (*U* = 5, *p* = 0.021) with an effect size of *r* = −0.48. No such effect was observed in healthy relatives or controls. Furthermore, no significant influence of any other SNP on the SOA was found.

## Discussion

We found an association of the cholinergic nicotinic receptor gene alpha 7 subunit (CHRNA7) with schizophrenia. Particularly one SNP (rs904952) in intron 4 seems to be important: T allele carriers were more often among schizophrenic patients compared to healthy controls in the German sample. Similar results were found in the Georgian sample but data did not reach significance, probably due to the small sample size. Gault et al. (49) showed that six different CHRNA7 transcripts are generated in the human brain by alternative splicing of exons 3 through 5. This could be at least partly directed by sequence variations in intron 4, explaining the major effect of the genetic variations in our study. Our results further support the significance of the nicotinic system as an important pathway in schizophrenia which may be the target for pharmacological intervention.

Our findings are in line with the majority of prior studies in this field. Case-control (9–11) and family (13, 14, 50–53) studies found a positive relationship between polymorphisms in the CHRNA7 gene and schizophrenia. Most of these studies investigated microsatellites as genetic markers. At the moment of conducting the present study, there were no SNPs reported to be associated with schizophrenia. Later Stephens et al. (11) and Stephens et al. (54) found three SNPs, which were significantly associated witch diagnosis of schizophrenia. Fan et al. (55) conducted both case-control as well as family studies in a Chinese population and found no relationship between schizophrenia and D15S1360, the dinucleotide microsatellite marker previously identified in several association and linkage studies positively associated with schizophrenia. Iwata et al. (42) investigated 11 SNPs and three microsatellites, including D15S1360 and rs904952, in Chinese patients and controls and found no significant association between polymorphisms and schizophrenia. In addition, publication bias has to be taken into account.

It is known for more than half century that visual information processing is impaired in schizophrenic patients (25, 26, 30, 31, 56). Whereas performance deteriorates also in healthy observers, the deterioration is much more pronounced in schizophrenic patients (57–59). Visual masking deficits have been proposed for decades to be a vulnerability marker for schizophrenia (60–64). Evidence comes from studies showing that also unaffected siblings of patients show masking deficits compared to controls (65–68), likewise adolescents with psychosis adolescents (69–72), and that masking deficits are rather constant for more than year in schizophrenic patients (73, 74). A particularly sensitive masking paradigm is the shine-through effect, revealing spatio-temporal information processing at the brink of human visual resolution in healthy human observers (75, 76) and schizophrenic patients (27–31). In a previous study, we have shown that the shine-through paradigm is a particular sensitive VBM technique which outperforms classical cognitive tests such as the CPT and the WCST (VBM sensitivity: 87%, specificity: 89%) (27). Moreover, the shine-through masking paradigm meets the practicability and explicability criteria proposed by Turetsky et al. (77). It is easy and fast to apply, and independent of culture, education, and gender.

As in previous research (27), the present study also identified significant performance differences between patients, relatives, and controls in the Georgian sample. In addition, we found a robust association of SNP rs904952 with VBM, the same SNP that was also found to be associated with schizophrenia in the German sample. This finding supports the important role of the nicotinic system in the pathophysiology of schizophrenia and related clinical parameters such as VBM performance. It needs to be evaluated if drugs affecting the nicotinic system influence VBM.

In our study, the positive association between CHRNA7 and VBM was only found in patients but not in the healthy relatives as we may have expected it from a vulnerability marker. This result is likely due to the small sample size but may also be explained by additional genetic or non-genetic components not present in the relatives. With CHRNA7 we may have identified one important component in the pathophysiology of schizophrenia. However, other factors are certainly necessary. It is interesting, and very much at the heart of the endophenotype concept, that VBM deficits were significantly associated with mutations in the cholinergic system even though there was no significant association with the diagnosis of schizophrenia.

There are limitations with our study. None of the GWAS studies so far could confirm a single candidate gene for schizophrenia although statistical power of these studies is usually considerably larger than in conventional association studies (78, 79). Therefore, the association of CHRNA7 with schizophrenia needs to be carefully followed up. In addition, unknown confounding factors may lead to a hidden stratification bias. The significant SNP rs904952 is not in HWE in the German sample. This might be due to sampling procedures since inclusion criteria required only one generation of Central European descent prior to the participant. Particularly in Berlin with strong migration, this might have led to a partly inhomogeneous sample. Another limitation of our VBM study is the small sample size, although a significant effect with such a small sample size argues rather for the reliability of the findings. Still, the results on VBM need to be replicated in an independent and larger sample. In addition, studies are needed to investigate the expression of CHRNA7 transcripts in brains of schizophrenic patients to identify the specific risk domain in the gene. Goghari et al. found an association of backward masking deficits with the COMT Val158Met in schizophrenic patients (80). Hence, future research has to evaluate other promising candidate genes of schizophrenia and their common impact on VBM.

In summary we found a positive association between a polymorphism of the CHRNA7 gene and schizophrenia. In the smaller Georgian sample the same trend was observed. In line with prior studies, VBM with the shine-through masking paradigm showed profound deficits in schizophrenic patients and, to a lower extent, in their healthy first-degree relatives. In addition, we have shown that VBM has a genetic component that runs in parallel with the diagnosis of schizophrenia.

## Conflict of Interest Statement

The authors declare that the research was conducted in the absence of any commercial or financial relationships that could be construed as a potential conflict of interest.

## Footnotes

^1^http://marketing.appliedbiosystems.com/mk/get/snpb_landing

^2^http://www.genome.ucsc.edu/cgi-bin/hgGateway

^3^http://www.ncbi.nlm.nih.gov/sites/entrez
